# The role of habituation in hippocampus-dependent spatial working memory tasks: Evidence from GluA1 AMPA receptor subunit knockout mice

**DOI:** 10.1002/hipo.20896

**Published:** 2010-12-01

**Authors:** David J Sanderson, David M Bannerman

**Affiliations:** Department of Experimental Psychology, University of OxfordSouth Parks Road, Oxford, United Kingdom

**Keywords:** hippocampus, working memory, mice, habituation, plasticity, AMPA receptors

## Abstract

Spatial alternation, win-shift behavior has been claimed to be a test of working memory in rodents that requires active maintenance of relevant, trial-specific information. In this review, we describe work with GluA1 AMPA receptor subunit knockout mice that show impaired spatial alternation, but normal spatial reference memory. Due to their selective impairment on spatial alternation, GluA1 knockout mice provide a means by which the psychological processes underlying alternation can be examined. We now argue that the spatial alternation deficit in GluA1 knockout mice is due to an inability to show stimulus-specific, short-term habituation to recently experienced stimuli. Short-term habituation involves a temporary reduction in attention paid to recently presented stimuli, and is thus a distinct process from those that are involved in working memory in humans. We have recently demonstrated that GluA1 knockout mice show impaired short-term habituation, but, surprisingly, show enhanced long-term spatial habituation. Thus, GluA1 deletion reveals that there is competition between short-term and long-term processes in memory. © 2010 Wiley Periodicals, Inc.

## INTRODUCTION

One of the simplest forms of spatial learning that rodents readily display is the ability to spatially alternate their responses when exploring an environment. Thus, if an animal has previously explored a spatial location it will subsequently be less likely to explore this same location and will alternate its response so that it explores a different spatial location. This behavior may underpin successful foraging in the wild, by reducing the likelihood of returning to a location in which food sources have been depleted. In the laboratory alternation has been widely tested by using maze tasks in which rodents are required to alternate or “nonmatch” their responses to a previous response that they made. For example, rodents may be rewarded for choosing an arm of a maze that they had not most recently explored. This is often referred to as “win-shift” behavior. Although, there are many factors that contribute to alternation (e.g., Dudchenko,^2001^; Dudchenko and Davidson,^2002^), it appears to be predominantly caused by the use of extramaze, allocentric cues (Walker et al.,^1955^; Douglas,^1966^; Futter and Aggleton,^2006^). Furthermore, it has been suggested that alternation behavior is evidence of ‘spatial working memory’ in rodents (Olton et al.,^1979^).

Regardless of the specific demands of the task, it has been found that the hippocampal formation is essential for spatial alternation behavior (Roberts et al.,^1962^; Olton and Papas,^1979^; Rawlins and Olton,^1982^; Bannerman et al.,^1999^). A large proportion of the research that supports this claim has come from lesions studies. However, it is often not possible to determine the cause of any deficit in these tasks from the lesion studies. For example, the hippocampus may be necessary for representing the spatial location of the arms or it may be necessary for expression or retrieval of a spatial memory, or indeed, it may be important for both of these processes. Moreover, lesion studies provide little insight into the molecular and synaptic processes underlying alternation behavior. Recent work with genetically modified mice has, however, demonstrated a potential psychological process that controls the expression of spatial information that results in alternation behavior.

In this review, we will evaluate the claim that alternation behavior is a form of memory that is analogous to working memory in humans. Then we will discuss recent work with GluA1 knockout mice that suggests that alternation behavior in rodents is governed by a form of short-term, stimulus-specific habituation (Sanderson et al.,2009b). We apply these findings to a theory of learning that assumes that memory is controlled by both associative and nonassociative processes (Wagner,^1981^). We finally argue that so called “spatial working memory” tasks in rodents and working memory in humans may, in fact, reflect different psychological processes. This is an important issue for translational studies and animal models of human working memory dysfunction. However, by understanding the psychological processes governing alternation behavior in rodents we may be able to use these tasks to model certain aspects of attention, which may also be of relevance to certain psychiatric conditions.

## ALTERNATION AS A FORM OF WORKING MEMORY

Working memory in humans refers to aspects of attention and executive function in short-term memory (Baddeley and Hitch,^1974^; Baddeley,^1992^). It is seen as a distinct process in short-term memory, in that it reflects the ability to temporarily maintain and manipulate information so that it can be used in a flexible manner. Therefore, it does not merely reflect passive maintenance of memory traces. This can be demonstrated in tasks such as reverse digit span and the *n*-back task. In the backward digit span task the capacity of working memory is measured by the number of items that can be recalled. However, subjects have to provide the information in the reverse order to which it was presented. Thus, not only does the relevant information have to be retained over a period of time, the correct response requires manipulation of the information. In the *n*-back task subjects are presented with a series of items, some of which are repeated in the series. Subjects are required to make a response if the present item is the same as a previous item that occurred a certain number of places back in the list. For example, the correct response may be to respond to items that match stimuli that occurred two places back in the list (i.e., a two-back task). Therefore, not only are subjects required to maintain information from the list, but they are also required to use the information flexibly so that they make the appropriate response.

The ability to test working memory in nonhuman animals would be highly advantageous for testing models of disorders that have been found to affect working memory in humans, such as schizophrenia (Goldman-Rakic,^1994^), dementia (Morris and Baddeley,^1988^) and developmental disorders such as attention-deficit hyperactivity disorder (Barkley,^1997^). In the late1970s Olton et al. suggested that it is possible to measure working memory ability in rodents using certain, win-shift spatial learning tasks (Olton et al.,^1979^; Olton and Papas,^1979^). We will briefly describe Olton and colleagues' account of win-shift behavior in spatial learning tasks.

Olton et al. (1979) adopted terminology used by Honig (1978) to suggest that spatial working memory could be distinguished as a separate process from spatial reference memory. They suggested that whereas spatial working memory requires the ability to maintain trial-specific information for a limited period of time so that spatial stimuli can be responded to in a flexible manner, spatial reference memory requires the ability to learn the correct fixed response to a stimulus, because of a constant association between a stimulus and an outcome. For instance, the ability to solve a discrimination in which one spatial location is always paired with reward and another location is not requires reference memory, but the ability to alternate responding between spatial locations on the basis of whether they have already been visited on that trial requires working memory.

An example of a task thought to require working memory is the T-maze alternation task (e.g., Rawlins and Olton,^1982^). In this task an animal receives trials that consist of two phases: a sample phase and a choice phase. In the sample phase animals are required to run down the stem of a T-shaped maze and are then forced into either the left or right goal arm to gain a food reward. During the sample phase entry into the other arm is blocked. After the animal has consumed the food reward it is placed back on the stem of the T-maze and is given a free choice between the sampled goal arm (i.e., the arm previously entered) and the arm that they previously did not enter. Animals are rewarded for choosing the arm that they did not enter during the sample phase.

Typically, in the T-maze alternation task rodents are given a series of trials with an equal number of trials in which the sample arm is the left or right goal arm, in a pseudorandom order. Thus, the task requires animals to remember the stimuli encountered in the sample phase, but this information is only relevant to the current trial and will not be relevant for subsequent trials. Olton et al. (1979) suggested that the ability of animals to display this trial-specific memory demonstrates a form of working memory.

Olton et al. (1979) claimed that the optimal strategy for performance on the T-maze is to remember the sample arm information during the interval between the end of the sample phase and the start of the choice phase. However, this information should be forgotten at then end of the trial, because this trial-specific information is not relevant for the subsequent trial. Therefore, animals must remember the stimuli that were presented and also when they were presented so that they do not interfere with subsequent performance. From this account of T-maze alternation performance it can be seen that Olton and colleagues' view of working memory in rodents might be seen to equate with the description of working memory in humans. Thus, according to Olton et al. (1979) T-maze alternation requires the manipulation of temporarily stored information.

Another task that Olton and his colleagues used to examine spatial “working memory” is the radial-arm maze task (Olton and Samuelson,^1976^; Olton et al.,^1978^; Olton and Papas,^1979^). The maze consists of a central platform that has a number of arms (often 6, 8, or 12) radiating out from the center, like spokes of a bicycle wheel. On the working memory version of the task, at the start of a trial each arm of the maze is baited with food reward and animals are allowed to enter the arms to consume the reward. After the food has been eaten from an arm it is not replaced on that trial. Consequently, if an animal enters an arm of the maze that it has previously visited within the trial then no food will be present. Thus, the efficient strategy is to avoid re-entering arms so that all the food rewards can be collected in as few choices as possible. Similar to the T-maze task this requires alternating or nonmatching with respect to a previous choice (or choices). Demonstration of efficient searching on the maze would imply that animals have a memory for where they have previously been, but obviously this information would not be relevant for subsequent trials.

Once again, Olton et al. (1979) emphasized that nonmatching-to-place in the radial-arm maze is an example of working memory because it requires active maintenance of memory within a trial, but to avoid interference between trials, information should be forgotten at the end of the trial. This idea assumes that nonmatching-to-place does not merely reflect passive memory for spatial locations, because it requires active manipulation of the information such that it can be used flexibly.

## THE ROLE OF THE HIPPOCAMPUS IN ALTERNATION BEHAVIOR

Lesions of the hippocampus profoundly affect performance on both T-maze alternation and win-shift, nonmatching-to-place behavior in the radial arm maze (Olton and Papas,^1979^; Rawlins and Olton,^1982^; Bannerman et al.,^1999^). Complete lesions of the hippocampus typically result in animals showing chance performance on these tasks (e.g., Bannerman et al.,^1999^). While these findings demonstrate that the hippocampus is necessary for spatial working memory, it is not possible to determine whether lesions specifically impair working memory ability or whether lesions impair the ability to form or utilize a representation of space, or indeed, both. For example, in rodents hippocampal lesions impair the ability to learn that a spatial location is paired with food reward, and also to associate a spatial location with escape in the Morris watermaze task (Morris et al.,^1982^; Deacon et al.,^2002^). In these spatial reference memory tasks hippocampal lesioned animals fail to discriminate correct from incorrect spatial locations. This has been taken as evidence for the claim that the hippocampus is necessary for representing space rather than being necessary for working memory (O'Keefe and Nadel,^1978^). Therefore, hippocampal lesions, while demonstrating the necessity of the hippocampus for alternation behavior, fail to reveal the specific neural substrates and psychological processes underlying spatial working memory.

Recently, the use of genetically modified mice that lack the GluA1 subunit of the AMPA receptor (Zamanillo et al.,^1999^) has shed new light on this issue. These GluA1 knockout (GluA1^−/−^) mice demonstrate that spatial alternation behavior can be dissociated from the ability to represent arrays of spatial cues. Before describing the pattern of behavioral impairments in GluA1^−/−^ mice we will briefly describe the biochemical and electrophysical consequences of GluA1 knockout.

## GLUA1 KNOCKOUT MICE

AMPA receptors are important for plasticity due to their role in postsynaptic depolarization, which allows for the neurotransmission of glutamate. AMPA receptors are necessary for tetanus induced NMDA-dependent long-term potentiation (LTP), because they allow the magnesium ion to be unblocked from the NMDA receptor. The AMPA receptor is made up of four subunits; GluA1–4. Our research has focused on the GluA1 subunit by testing genetically modified mice that lack GluA1 on learning and memory tasks. The GluA1 (GluR-A, GluR1) knockout mouse is a constitutive knockout in which functional GluA1 genes (*gria 1*) are absent in all cells, including those in the brain, throughout the entire lifetime of the animal (Zamanillo et al.,^1999^). The expression levels of other glutamate receptor subunits (e.g., GluA2, GluA3, GluN1, GluN2A), however, remain unchanged. GluA1^−/−^ mice exhibit normal development, life expectancy and fine structure of neuronal dendrites and synapses. They do, however, exhibit a marked reduction in the number of functional AMPA receptors. What AMPA receptors are available are preferentially targeted to synapses. Thus, soma-patch currents recorded in CA1 pyramidal cells are strongly reduced. In the original description of these mice, it was reported that glutamatergic synaptic currents were largely unaltered (Zamanillo et al.,^1999^), although in subsequent studies it was shown that, in fact, the AMPA receptor-mediated synaptic currents were also reduced in GluA1^−/−^ mice (Andrasfalvy et al.,^2003^; Jensen et al.,^2003^). For example, the mean CA1 field EPSP in GluA1^−/−^ mice, in response to a single test pulse of 70 μA, was only 65% of the mean field EPSP in wild-type mice (Romberg et al.,^2009^). Thus, excitatory synaptic transmission is attenuated in these animals.

Deletion of GluA1 also affects hippocampal synaptic plasticity. Although the induction of hippocampal long-term potentiation (LTP), the most commonly studied form of hippocampal synaptic plasticity, requires the NMDA subtype of glutamate receptor (Collingridge et al.,^1983^), the continued expression of LTP depends, at least in part, on the translocation of additional AMPA receptors into the postsynaptic membrane (for reviews see Malinow and Malenka,^2002^; Kessels and Malinow,^2009^). In turn, this activity-dependent insertion of AMPA receptors may depend, in part, on the GluA1 subunit (Shi et al.,^2001^). Although the mechanisms underlying the delivery and insertion of GluA1-containing AMPA receptors into the postsynaptic membrane is not fully understood, it is thought to involve the phosphorylation of key amino acid residues on the GluA1 subunit.

Consistent with this putative role for GluA1-containing AMPA receptors in the strengthening of synaptic connections, initial electrophysiological characterization of GluA1^−/−^ mice showed that hippocampal long-term potentiation (LTP), induced by high frequency tetanic stimulation, was abolished at Schaffer collateral—CA1 pyramidal cell synapses during in vitro recordings made in slice preparations from adult animals (Zamanillo et al.,^1999^). However, more recent studies have revealed different results. For example, Frey et al. (2009) recently showed that spike-timing dependent plasticity in CA1 pyramidal cells is GluA1-independent. Furthermore, studies using a θ-burst induction paradigm, revealed a gradually developing form of LTP in the knockouts (Hoffman et al.,^2002^; Romberg et al.,^2009^). In these studies, the amount of LTP in the GluA1^−/−^ mice was found to be indistinguishable from that in the wild-types, 20 to 45 min after θ-burst induction (but see Erickson et al.,^2010^). Thus, following θ-burst stimulation, at least, GluA1 appears to contribute more to the early, rapidly decaying component of LTP, which might actually be considered more akin to a form of short-term potentiation (STP). Consistent with these findings, Erickson et al. (2010) have recently shown that weaker stimuli, which are insufficient to induce LTP, result in STP in wild-type mice, and that this is greatly reduced in the GluA1^−/−^ mice.

It is widely thought that synaptic plasticity in the hippocampus is necessary for spatial learning (e.g., Morris et al.,^1986^; Martin et al.,^2000^). Given the effects of GluA1 deletion on synaptic plasticity it might be predicted that that deletion of the GluA1 AMPA receptor subunit should result in a spatial learning deficit. However, GluA1^−/−^ mice show normal reference memory in the Morris water maze (Zamanillo et al.,^1999^; Reisel et al.,^2002^). They also show normal spatial reference memory on appetitively motivated tasks (Reisel et al.,^2002^; Schmitt et al.,^2003^). This is in contrast to hippocampal lesioned mice that fail to learn these tasks. Therefore, GluA1^−/−^ mice, despite reduced hippocampal synaptic plasticity, are able to discriminate spatial stimuli and respond appropriately in order to obtain food or find escape from water, thus indicating normal learning of associations between spatial locations and rewards.

## GLUA1 AND SPATIAL WORKING MEMORY TASKS

In contrast to their normal performance on spatial reference memory tasks, GluA1^−/−^ mice show impaired alternation (nonmatching-to-place) behavior. Although wild-type control mice show a high level of performance on the T-maze alternation task, GluA1^−/−^ mice fail to rise above chance levels (i.e., 50% alternation, Reisel et al.,^2002^). This suggests that the ability to represent spatial locations and to discriminate between them on the basis of their association with different outcomes (i.e., spatial reference memory) can be dissociated from the ability to demonstrate memory for recently visited spatial locations in an alternation task (i.e. spatial working memory).

**Figure 1:**
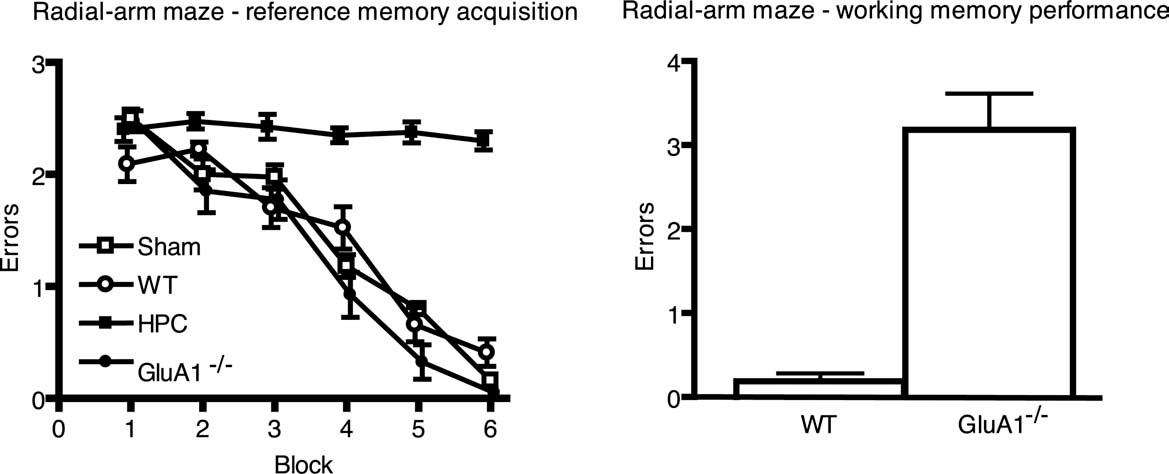
Hippocampal lesions, but not GluA1 knockout impair acquisition of the spatial reference memory component of the radial maze task. Radial-arm maze—reference memory acquisition: mean number of reference memory errors per trial (±SEM) during six blocks of training (four trials per block). Mice were trained to discriminate between three baited arms and three nonbaited arms of a six-arm radial-arm maze. Doors prevented mice re-entering arms they had already visited on that particular visit to the maze (i.e., prevented working memory errors) during this stage of the experiment. Sham lesioned (Sham), wild-type (WT), and GluA1^−/−^ mice all acquired the task at a similar rate. Hippocampal lesioned mice (HPC) were completely unable to learn which arms were baited and which arms were not baited. Radial-arm maze—working memory performance: mean number of working memory errors per trial (±SEM) for wild-type (WT) and GluA1^−/−^ mice. During this stage of testing mice were still rewarded in the same three arms of the maze and not rewarded in the three nonbaited arms, but now they were allowed to re-enter arms as often as they liked, and rewards were not replaced within a trial. Reproduced with permission from Schmitt et al., J Neurosci,^2003^, 23, 3953–3959, © Society for Neuroscience.

This dissociation is most clearly demonstrated in an experiment that simultaneously tested spatial discrimination learning and spatial alternation behavior within the same task (Schmitt et al.,^2003^). In this task, during the training phase mice were required to learn that three arms of a six-arm radial-arm maze were baited with food reward, while the remaining three arms were not. After having entered an arm mice were allowed to return to the center of the maze and the door to the arm was closed. This occurred regardless of whether the arm was baited or not. Thus, within a trial, mice could not enter an arm more than once, and so mice could not make “working memory errors.” Therefore, performance during the training phase reflects only spatial reference memory. In contrast to hippocampal lesioned mice that failed to learn this task, GluA1^−/−^ mice learnt the task at a similar rate as control mice (Schmitt et al.,^2003^ see Fig. 1).

Then in a test session at the end of training mice were allowed collect all the food rewards, but now mice could make repeated entries into the arms of the maze. Re-entering a previously visited arm was classed as a “working memory error” because efficient performance relies on animals keeping track of where they have been. Figure 1 shows the number of working memory errors that the wild-type and GluA1^−/−^ mice made during this phase of testing. Although control mice made very few working memory errors during this test, GluA1^−/−^ mice made a significantly greater number of errors. The two groups however, still did not differ in the number of reference memory errors (Schmitt et al.,^2003^).

The data from GluA1^−/−^ mice suggest that there are two dissociable forms of spatial information processing, both of which depend on the hippocampus for their expression. There is a GluA1-dependent form of information processing, which underlies, or at least contributes to, alternation in the T-maze and win-shift behavior in the radial-arm maze. Then there is a GluA1-independent mechanism that is required for forming associations between spatial locations and outcomes. Thus, in contrast to hippocampal lesions, the GluA1^−/−^ mouse provides a means of examining the psychological mechanisms underlying spatial working memory that is independent of the ability to learn spatial reference memory tasks. While it is tempting to think that GluA1 is necessary for spatial working memory as described by Olton et al. (1979), and thus provides a model of working memory disorders in humans, it is important to consider alternative explanations for alternation behavior in rodents.

## ALTERNATION AND WORKING MEMORY

**Figure 2:**
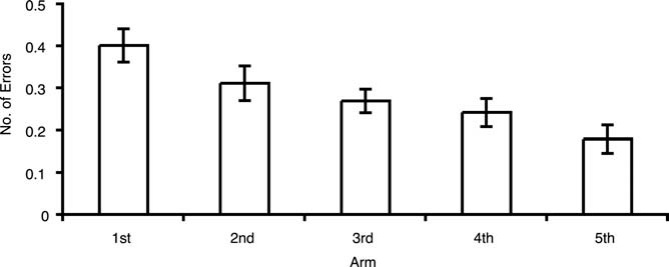
Delay-dependent working memory performance in C57BL/6 mice. The mean number of working memory errors (±SEM) made between the fifth and sixth, final correct choice on a six-arm radial maze as a function of the order in which the arms were originally visited. Data averaged across 12 trials. Mice were more likely to make errors to arms of that were less recently visited than arms that were more recently visited (significant linear trend *F*_(1,23)_ = 38.21, *P* < 0.0005, unpublished data).

Alternation has been viewed as a test of working memory because the two phenomenon share similar characteristics. For example, animals flexibly respond to stimuli on the basis of trial-specific information. Also, the retention of information is susceptible to disruption by delays and interference. This is illustrated by the performance of mice on a working memory version of the radial-arm maze (unpublished observations, see Sanderson et al.,2009a). C576BL/6 mice received 12 training trials on a six-arm radial-arm maze task in which they were allowed to enter arms until they had consumed all six rewards. Figure 2 shows the number of incorrect arm entries (i.e., working memory errors) that mice made, specifically between the fifth and the sixth, final correct choice as a function of the temporal order of the arms as they were originally chosen within a trial. Across the five arms there is a linear trend showing that mice made more errors to arms entered earlier within the trial compared to arms entered later in the trial, thus implying greater memory of the recently experienced arms. These data are consistent with the idea that working memory requires temporary maintenance of information and is sensitive to interference caused by either the temporal delay between the sample and choice, or the experience of other spatial stimuli between sample and choice. While this evidence may provide alternation with some face validity as a test of working memory, it is also possible to explain the behavior in simpler terms that do not assume active maintenance and manipulation of information.

## IS THE ABILITY TO ALTERNATE INNATE OR ACQUIRED?

One of the assumptions of the working memory account of alternation is that to be able to demonstrate working memory the animal has first had to learn the alternation, win-shift, or nonmatching-to-place rule. Thus, an animal should learn that if food has been consumed in a particular spatial location within a trial, then it should avoid returning to that location. The rule only applies within trials, not between trials. It would be expected that if rodents are capable of learning such a rule then it would be learnt gradually by trial and error. Notably rodents are often well above chance at the start of testing on tasks such as the T-maze alternation task and nonmatching to position in the radial-arm maze (e.g., >80% correct, Bannerman et al.,^1999^). Thus, it appears that animals can demonstrate alternation without necessarily having to acquire a rule. Also, when performance does improve with training, rather than being a demonstration of rule acquisition, this may merely reflect the relative ability in which spatial locations are discriminated (e.g., a perceptual learning effect, Trobalon et al.,^1991^).Therefore, it is not certain that animals have acquired the rule. Importantly, rodents spontaneously alternate without reinforcement for alternating. In the spontaneous alternation task, the animal is allowed to enter one of the goal arms of a T-maze during a sample trial and explore for a short period of time (e.g., for 30 s). It is then removed from the maze and, after a short delay (e.g., 15 s), it is returned to the maze and given a free choice of either arm. Animals are given a number of these trials and the number of alternations is calculated. Normal mice show a strong preference to alternate, even in the absence of any food rewards in the arms. Thus, it appears that animals have an innate preference to alternate that need not be acquired. Therefore, alternation may reflect a simpler psychological process than rule learning. Importantly, spontaneous alternation, like rewarded alternation and win-shift behavior on the radial-arm maze, is sensitive to both hippocampal lesions (Deacon et al.,^2002^) and also to GluA1 deletion (Bannerman et al.,^2004^; Sanderson et al.,^2007^).

## ALTERNATION AS A FORM OF SHORT-TERM STIMULUS-SPECIFIC HABITUATION

A different account of alternation behavior is that it reflects short-term habituation to spatial stimuli. Habituation describes the phenomenon in which there is reduction in unconditioned responding to repeated presentations of a stimulus. For example, the first presentation of a stimulus may elicit an unconditioned response (UR), but this UR will decline or habituate with repeated presentations. Therefore, if it is assumed that spatial stimuli elicit an unconditioned exploratory response, this exploratory response will decline as spatial stimuli become familiar. In a test of spontaneous alternation, whereas the sample arm may be capable of eliciting exploration on its first presentation, on the choice trial habituation should have occurred such that the sampled arm will be less likely to elicit exploration. Habituation will, of course, not have occurred to the nonsampled arm. Thus, the novel (nonsampled arm) will be able to generate greater levels of exploration than the previously sampled arm, and animals will show alternation behavior. Therefore, alternation can be described as a demonstration of stimulus-specific habituation. If we assume that this habituation effect occurs every time a spatial location is experienced and is short lasting, then it is clear that habituation can account for why animals show correct performance within a trial of T-maze alternation and nonmatching-to-place in the radial-arm maze, and why they continue to show correct performance across trials. For example, in the T-maze alternation task, across training, both goal arms are likely to have become familiar, but however, on a choice trial, the sample arm will be relatively more familiar than the nonsampled arm that has been less recently visited. Therefore, because of the greater relative level of short-term habituation to the more recently visited, sample arm animals should show greater levels of exploration to the less recently visited goal arm, and thus alternate their response. The key difference between the description of alternation as habituation and alternation as working memory is that the habituation account does not assume that performance relies on active maintenance and manipulation of information. The habituation account merely assumes that passive experience of stimuli results in a psychological process that causes a reduction of responding to those stimuli.

## SPATIAL NOVELTY PREFERENCE

**Figure 3:**
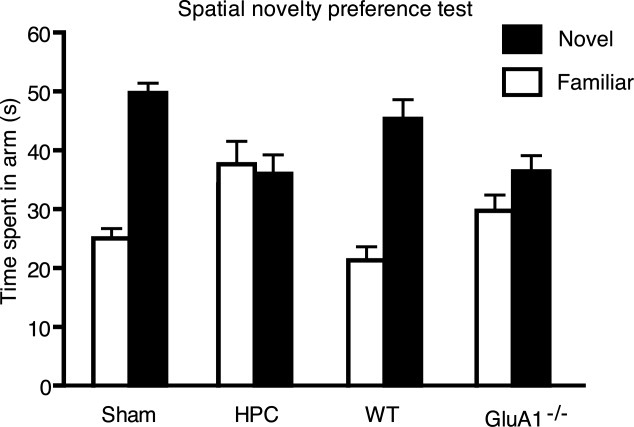
Both hippocampal lesions and GluA1 knockout impair performance on a spatial novelty preference test. Mean time spent in arms (±SEM). Sham and wild-type (WT) mice exhibit a preference for a previously unexposed (Novel) arm of a Y-maze over a familiar arm to which they have previously been exposed. GluA1^−/−^ mice and hippocampal lesioned mice (HPC) did not show a significant preference for the novel arm. Reproduced with permission from Sanderson et al., Behav Neurosci,^2007^, 121:559–569, © American Psychological Association.

Support for the hypothesis that GluA1 deletion impairs habituation comes from a study that examined spatial novelty preference in mice (Sanderson et al.,^2007^). In this task mice were exposed to two arms (start and familiar arms) of a Y-shaped maze for a period of 5 min (sample phase). During this time mice could explore the two arms of the maze such that the allocentric cues associated with the arms became familiar. After a 1-min interval mice were returned to the maze and were now allowed to explore the two familiar arms and a novel arm (test phase). If the previous exposure to the two familiar arms in the sample phase resulted in habituation of exploration, then mice should show a greater preference for exploring the novel arm over the familiar arms. This was found to be the case. Figure 3 shows the amount of time spent in the novel and familiar arms during the test phase. Whereas, wild-type mice showed a preference for the novel arm, GluA1^−/−^ mice did not and were impaired relative to controls. This is consistent with the idea that GluA1 is necessary for short-term habituation, (Sanderson et al.,^2007^ see Fig. 3).

This spatial novelty preference task used by Sanderson et al. (2007) is a test of stimulus-specific habituation to spatial cues. It is worth noting that the design of the task is analogous to that of object recognition (Ennaceur and Delacour,^1988^) in which animals are exposed to two identical copies of an object (i.e., A1 and A2) and then after an interval are allowed to explore a third copy of the familiar object (A3) and a novel object (B1). Animals typically show a preference for exploring the novel object, thus indicating that exploration of the familiar object has habituated. Interestingly, while repeated presentations of a stimulus produce a reduction in unconditioned responding, it has been found that neuronal activity is reduced to repetitions of stimuli (Brown et al.,^1987^; Brown and Aggleton,^2001^; Henson et al.,^2003^; Ranganath and Rainer,^2003^; Grill-Spector et al.,^2006^; Kumaran and Maguire,^2006^). It is possible that GluA1 may play a part in the neuronal processes responsible for this repetition suppression effect.

## WAGNER's ACCOUNT OF HABITUATION

Wagner (1976,1981) proposed that habituation is caused by a representation of a stimulus being active in a refractory memory state at the time when the stimulus is presented. A representation can become active as a result of either a recent presentation of the stimulus or by associative retrieval of the representation. These separate nonassociative and associative processes result in short-term and long-term habituation, respectively. We will briefly provide a description of Wagner's theory, before describing how the theory can be used to make novel predictions regarding the role of GluA1 in habituation.

**Figure 4:**
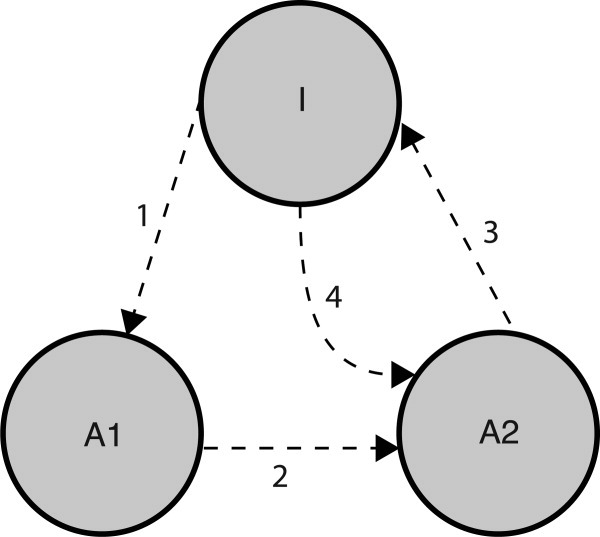
The states of activation, in which elements of a memory can reside, and the permissible transitional routes between states, according to Wagner (1981). Presentation of a stimulus leads to a proportion of its elements transferring from the inactive state (I) to the A1 state (route 1). Elements rapidly decay to a secondary activation state, A2 (route 2), before eventually returning to an inactive state, I, (route 3). Elements that are active in the A2 state cannot return to the A1 state on subsequent presentation of the stimulus, thus leading to reduced A1 activity. A2 state activity can occur due to the recent presentation of a stimulus (self-generated priming; route 2). Also, presentation of a stimulus leads to A2 state activation of elements of other stimuli with which it is associated (retrieval-generated priming; route 4).

Wagner (1981) proposed that a stimulus is represented by a set of elements. When a stimulus is presented it is able to activate a proportion of its elements into a primary activity state (A1, see Fig. 4). Elements rapidly decay from the A1 state into a secondary activity state (A2) where they remain before gradually decaying back to an inactive state (I). While proportions of elements can be in the different activity states, individual elements can only be in one state at any one time. Whereas elements in the A1 state can generate strong levels of responding, elements in the A2 state cannot and are less able to generate responding. Also, whereas elements in the A1 state can receive processing, elements in the A2 state cannot. Thus associations can form between elements of different stimuli that are concurrently active in the A1 state, but not in the A2 state. If elements are in the A2 state when the stimulus is presented they are not able to return to the A1 state. Consequently, there is a reduction in the number of elements that are available to be activated into the A1 state, which results in a reduction in responding. Thus, habituation occurs to the degree to which the elements of the stimulus are in the A2 state. Habituation can occur simply because a recent presentation of a stimulus results in its elements being active in the A2 state (self-generated priming, see Fig. 4). However, if enough time has passed after a stimulus presentation, such that a stimulus' elements have returned to the inactive state, then habituation will not occur, because the stimulus will be able to fully activate its elements into the A1 state. Therefore, a recent presentation of a stimulus results in short-term habituation.

Whereas short-term habituation reflects a time-dependent decay process, long-term habituation reflects an associative retrieval process. Wagner (1981) suggested that long-term habituation occurs because associations formed between stimuli can result in elements being directly activated into the A2 state (retrieval-generated priming, see Fig. 4). Thus, if a stimulus (e.g., X) has formed an association with the context in which it is presented, then presentation of the context can associatively activate X's elements into the A2 state. Associative activation of elements into the A2 state results in a long-term form of habituation, because rather than being dependent on how recently a stimulus was experienced (as is the case for short-term habituation), the level of A2 activation is dependent on the strength of the association that was formed between stimuli.

The impairments on alternation performance suggest that short-term, nonassociative memory processes may be disrupted in GluA1^−/−^ mice. In contrast, the preserved ability of GluA1^−/−^ mice to form associations involving spatial stimuli on reference memory tasks (e.g., spatial location and food reward, or spatial location and escape from water, Zamanillo et al.,^1999^; Reisel et al.,^2002^; Schmitt et al.,^2003^), suggests that long-term memory is preserved in these mice. If this is the case then GluA1 deletion should impair short-term habituation, but spare long-term habituation.

## GLUA1 KNOCKOUT IMPAIRS SHORT-TERM SPATIAL HABITUATION AND ENHANCES LONG-TERM SPATIAL HABITUATION

**Figure 5:**
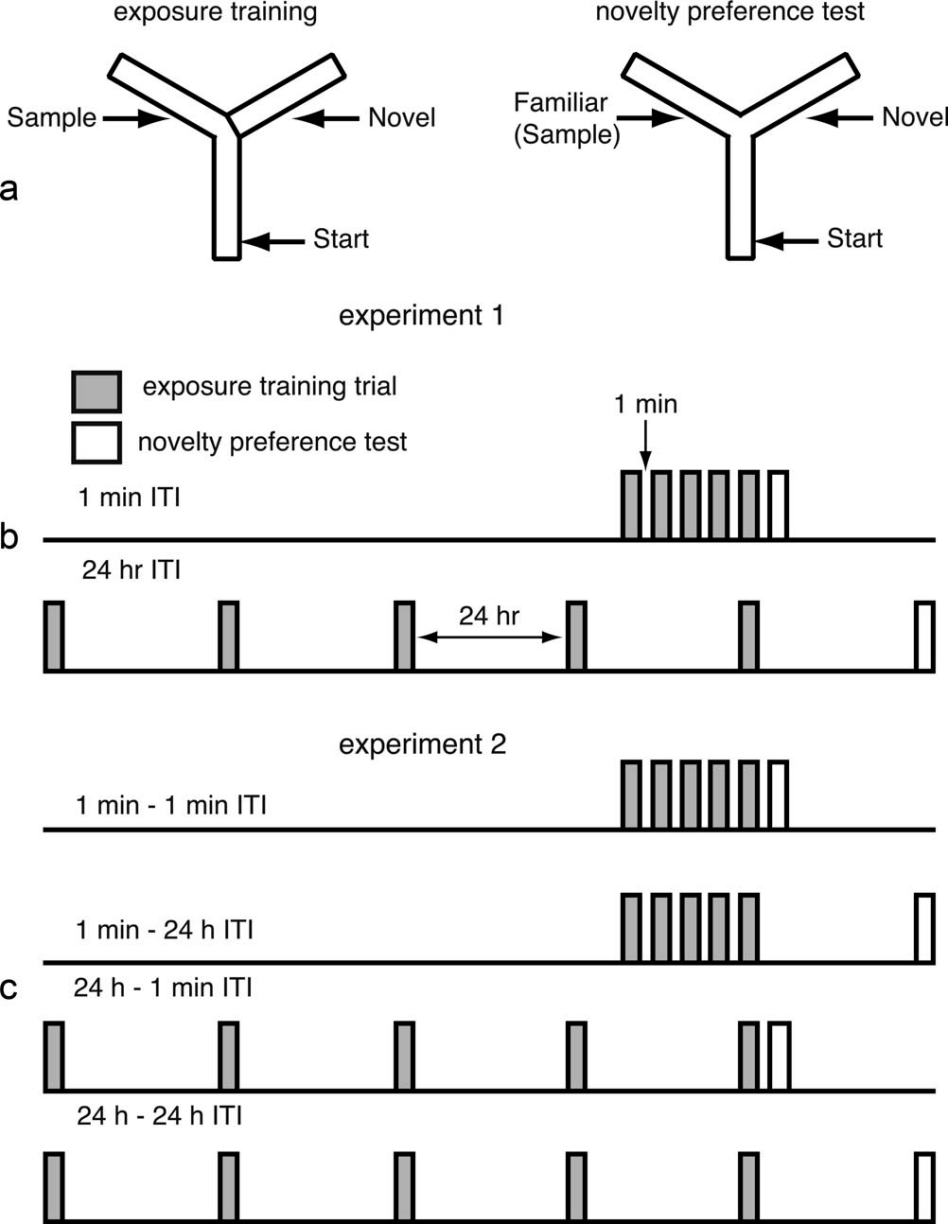
The design of Experiments 1 and 2 in Sanderson et al., (2009b). a, During exposure training mice were allowed to explore the Start arm and the Sample arm for five 2-min trials. Access to the Novel arm was blocked. During the novelty preference test mice were allowed to explore the two familiar arms (Start and Sample) and the previously unvisited, Novel arm for a period of 2 min. b, In Experiment 1, the interval between exposure trials (represented by the gray bars) and also the interval before the novelty preference test (represented by the white bars) was either 1 min (1 min ITI) or 24 h (24 h ITI). c, In Experiment 2, two groups of mice from each genotype received exposure training with a 1-min interval between trials and two further groups from each genotype received exposure training with a 24-h interval between trials. One group from each training condition received the novelty preference test 1 min after the last training trial. The other group received the test 24 h after the last training trial.

In a recent study, we tested the effects of GluA1 deletion on short-term and long-term habituation. The spatial novelty preference task was modified so that mice received repeated exposure training trials before receiving a novelty preference test (see Fig. 5a). Short and long-term habituation to spatial cues were assessed by manipulating (i) the length of the interval between the series of exposure training trials and (ii) the interval prior to the test of spatial memory (Sanderson et al.,2009b).

In the first experiment, mice received five 2-min exposure training trials to two arms of the Y-maze (the start and sample arms; see Fig. 5b), before a novelty preference test during which they could now choose between all three arms (i.e., the two familiar arms and a novel, unexposed arm, Sanderson et al.,2009b). In one condition, there was (i) a 1-min intertrial interval (ITI) between each of the training trials, and (ii) a 1-min interval between the last training trial and the test trial. In the other condition, the gap between each of the training trials, and between the last training trial and the preference test was 24 h (see Fig. 5b). When the short, 1 min ITI is used, performance should be maximally influenced by short-term memory processes. However, when the long, 24 h ITI is used, performance should reflect long-term memory.

**Figure 6:**
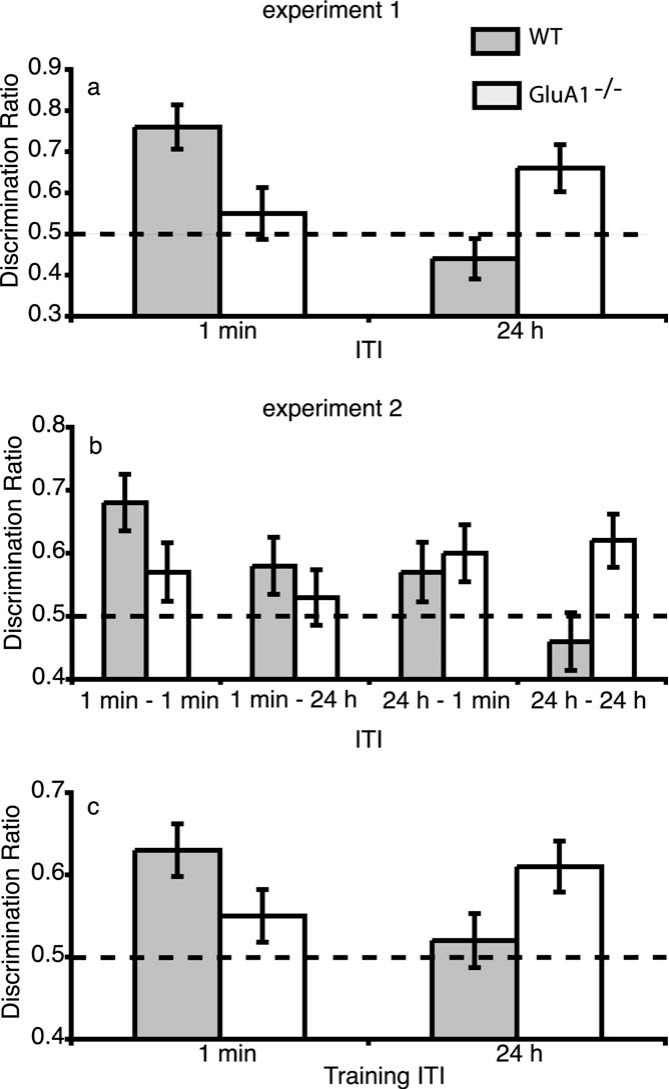
GluA1 knockout impairs short-term spatial novelty preference, but enhances long-term novelty preference. Time spent exploring the Novel arm is shown as a ratio of the combined time spent exploring the Novel and Familiar (Sample) arms during the test (i.e., discrimination ratio = Novel/[Novel + Familiar]). Scores greater than 0.5 indicate a novelty preference. The dashed lines indicate chance performance. Error bars indicate ±SEM. a, In Experiment 1 (see Fig. 5b) GluA1^−/−^ mice were impaired in the short, 1 min ITI condition, but enhanced in the long, 24 h ITI condition relative to wild-type mice (WT). b, In Experiment 2 (see Fig. 5c) GluA1^−/−^ mice were impaired when training trials were separated by a short 1-min interval, but enhanced when the training trials were separated by 24 h. There was no significant interaction between genotype and the test interval. c, The results of Experiment 2 collapsed across the short, 1 min and long, 24 h test intervals to show the independent effects of the training ITI in wild-type and GluA1^−/−^ mice. Reproduced with permission from Sanderson et al., Learn Mem,2009b, 16, 379–386, © Cold Spring Harbor Laboratory Press.

During the novelty preference test wild-type mice showed a strong preference to explore the novel arm in the 1 min ITI condition, whereas GluA1^−/−^ mice did not show this preference. However, in the 24 h ITI condition, GluA1^−/−^ mice actually showed a stronger novelty preference than the controls (Fig. 6a). The first result is consistent with the prediction that GluA1 is necessary for short-term habituation, but, surprisingly, we found that GluA1 deletion actually significantly enhanced long-term habituation.

The interval between exposure training trials and the interval prior to test may both have an independent effect on the novelty preference. Whereas the interval between exposure training trials may affect the rate of learning, the interval prior to the test may affect the expression of memory. Therefore, GluA1 deletion could differentially affect acquisition of learning that occurs in the massed (1 min ITI) and spaced (24 h ITI) conditions. Alternatively, GluA1 deletion may differentially influence the expression of memory, either across short (1 min), or long (24 h) intervals. To test the effect of these two factors, both the interval between exposure training trials and the interval before the novelty preference test were manipulated in a further experiment, adopting a between-subjects factorial design (see Fig. 5c).

Mice received exposure training with trials separated by either a 1 min or a 24 h ITI, as in the previous experiment (Sanderson et al.,2009b). After exposure training, half the mice from each ITI condition then received the novelty preference test after 1 min, and the remaining mice received the test after 24 h (see Fig. 5c). Thus, wild-type and GluA1^−/−^ mice were run in one of four groups. In agreement with experiment 1, when the training ITI was short (1 min) the GluA1^−/−^ mice showed a weaker novelty preference than controls, but GluA1^−/−^ mice showed a stronger novelty preference than controls when the training ITI was 24 h (Figs. 6b,c). However, this pattern was not influenced by the interval between the last training trial and the novelty preference test. Thus, the enhanced spatial recognition memory in the GluA1^−/−^ mice was the result of the interval between the training trials and not the result of the interval prior to the novelty preference test, thus demonstrating enhanced learning and not simply enhanced performance or expression of memory.

Importantly, both short-term and long-term spatial habituation were disrupted by hippocampal lesions (Sanderson et al.,2009b). In the novelty preference test, lesioned mice failed to show a novelty preference with either short or long ITIs. Therefore, the enhanced long-term spatial memory in GluA1^−/−^ mice reflects memory supported by the hippocampus.

This facilitation of long-term spatial memory in GluA1^−/−^ mice is an important result for a number of reasons. The most striking of these is that it actually demonstrates enhanced spatial memory in animals lacking a form of hippocampal synaptic plasticity. Furthermore, several accounts of the short-term spatial memory deficit in GluA1^−/−^ mice can be ruled out with this data set. First, the deficit does not occur because mice fail to discriminate between the arms of the maze. The GluA1 knockout mice showed successful discrimination of the arms of the maze (see also Zamanillo et al.,^1999^; Reisel et al.,^2002^; Schmitt et al.,^2003^), and also that they could do so on the basis of novelty preference. Second, any simple nonspecific effects of GluA1 deletion on locomotor activity or motivational states cannot explain these time-dependent effects on learning. No possible confound could cause the diametrically opposite effect on short-term and long-term learning using the same measures of performance. Importantly, two different performance measures (time in arms and number of arm entries) both demonstrated the same pattern of results (Sanderson et al.,2009b).

The facilitation of spatial memory observed with long interval training also argues against a simple, partial degradation of hippocampal function in GluA1^−/−^ mice. For example, it is possible that the pattern of impaired short-term spatial memory, but spared long-term spatial memory observed in these mice could reflect a nonspecific, but incomplete, disruption of hippocampal function, with short-term memory (or spatial working memory) performance simply being the more sensitive measure of hippocampal function. The demonstration of facilitated long-term spatial memory in GluA1^−/−^ mice argues strongly against this possibility. Furthermore, recent work by Rust et al., (2010) has provided some evidence that the actin filament depolymerizing protein, n-cofilin, is necessary for learning associations between spatial locations and outcomes, but not for short-term habituation to spatial stimuli. Taken together with the results with GluA1^−/−^ mice, the findings from Rust et al., (2010) suggest a double dissociation between associative and nonassociative processes in spatial learning (see Bannerman and Sprengel,^2010^).

The results of Sanderson et al. (2009b) do not fit with a simple trace decay interpretation of memory. Furthermore, they argue against a model whereby short-term memories are serially converted into long-term memories. This may have important implications for the relationship between AMPA receptor trafficking and different memory states (Malinow and Malenka,^2002^; Kessels and Malinow,^2009^).

The data argue for two dissociable memory processes. The fact that GluA1 deletion was able both to impair short-term spatial memory, and enhance long-term memory suggests that these forms of memory depend on separate psychological processes that can, under some circumstances, compete with one another. Thus, these results also do not support a single process account of habituation (Horn,^1967^; Mackintosh,^1987^), but are consistent with a dual-process memory model (Wagner,^1976^; Wagner,^1981^; Sanderson and Bannerman,^2010^).

Although spaced exposure training led to a spatial novelty preference in GluA1^−/−^ mice, these conditions led to poor performance in wild-type mice. This suggests that long-term, associative processes made little contribution to habituation in wild-type mice. One possible reason for this is that there was insufficient training for associative, long-term processes to develop. Thus, an increase in exposure training my increase the beneficial effect of spaced training in normal mice. We have recently shown this to be the case (Sanderson and Bannerman,^2010^). It was found that spaced training led to greater habituation than massed training, and contributed to a long-term habituation effect that was determined by the number of exposure training trials.

## A DUAL PROCESS ACCOUNT OF HABITUATION

As described in Wagner's Account of Habituation, Wagner (1976,1981) suggested that there are independent short-term and long-term memory processes that can both contribute to habituation. He suggested that short-term habituation reflects nonassociative, self-generated priming of the memory of a recently presented stimulus. In contrast, long-term habituation is thought to reflect associative, retrieval-generated priming of the memory for a stimulus. Long-term habituation is thus based on associations formed between the target stimulus and contextual cues, and therefore, the extent of the habituation is dependent on the strength of these associations. For example, in the Y-maze spatial novelty preference task exploration of the arms may lead to the formation of associations between cues such that associative retrieval may result in long-term habituation.

The pattern of impaired short-term habituation and enhanced long-term habituation in GluA1^−/−^ mice suggests that short-term and long-term processes compete with one another (Sanderson and Bannerman,^2010^). This idea can be accommodated by Wagner's (1981) theory. A feature of associative learning is that increments in long-term learning are greater when the occurrence of the stimuli are surprising (Rescorla and Wagner,^1972^; Wagner,^1981^). Of course, habituation renders stimuli less surprising. Therefore, a short-term memory of a stimulus can retard subsequent associative learning by rendering the occurrence of the stimuli involved unsurprising, and thus reducing the levels of attention that are subsequently paid to that stimulus (Wagner,^1981^). For example, Sunsay, Stetson and Bouton (2004) have shown that the recent presentation of a stimulus (CS1) impairs the ability of that stimulus to enter into associations with other stimuli when subsequently paired (CS1-US). This retardation in conditioning was not seen if a different stimulus (CS2) preceded the CS1-US pairing. The impairment in conditioning is due to the fact that a stimulus-specific short-term memory of the target (CS1) reduces subsequent processing of that target cue and thus disrupts long-term, associative memory formation.

If it is assumed that GluA1 deletion enhanced long-term habituation by reducing the competition between short-term and long-term memory processes, then it must be assumed that a short-term process may have reduced long-term memory in control mice. It is possible that within a trial elements activated into the A1 state may have decayed into the A2 state. The accumulation of elements in the A2 state during a trial would reduce the amount of elements that are active in the A1 state, thus limiting the amount of associative learning that could take place between concurrently presented stimuli. Thus, an interpretation of our data is that GluA1 deletion affects transfer of representational elements into the short-term memory A2 state. Thus, GluA1 knockout mice may show impaired short-term habituation to spatial stimuli within a trial. In turn, this may increase the opportunity for further processing of cues (in the A1 memory state), thus enhancing associative long-term memory formation.

This hypothesis is not without precedent. Conditioning occurs more readily with a conditioned stimulus (CS) of intermediate length duration, compared to a longer CS duration (Smith,^1968^). This is because a longer CS may undergo greater short-term habituation (i.e., more of its representational elements are in the A2 state) by the time the unconditioned stimulus (US) is presented. Consequently, there would be less CS elements available in the A1 state that would be able to form an association with the US. According to our analysis, GluA1 deletion may impair short-term habituation and enhance long-term habituation because it affects the rate of transfer between the A1 and A2 states. Because stimulus elements would therefore remain in the A1 state for longer then there would be an increased opportunity for the formation of long-term associations. This increase in associative learning could then subsequently lead to greater retrieval-generated priming and hence stronger long-term habituation. It is possible that GluA1 deletion may facilitate long-term learning by reducing the negative within-trial effects of short-term memory. These predictions are consistent with simulations of Wagner's model (Sanderson et al.,^2010^).

What is clear is that the pattern of impaired short-term habituation and enhanced long-term habituation in GluA1^−/−^ mice cannot be explained in terms of working or episodic memory. For example, a failure to actively maintain and manipulate a representation would not explain why GluA1^−/−^ mice show an enhanced long-term spatial novelty preference. The pattern of effects in GluA1^−/−^ mice are thus more readily accommodated by Wagner's model which assumes that habituation is caused by the passive decay of memory traces that are activated either directly by the stimulus (i.e., self-generated priming) or associatively (i.e., retrieval-generated priming).

Given the specific role of GluA1 in short-term spatial habituation, it is tempting to speculate on a role for a rapidly induced, short-lasting, GluA1-dependent form of synaptic plasticity in this process. However, while most electrophysiological studies examining the role of GluA1 in synaptic plasticity have concentrated on its role in potentiation of pyramidal cell activity (Zamanillo et al.,^1999^; Hoffman et al.,^2002^; Romberg et al.,^2009^; Erickson et al.,^2010^), this may not be the most plausible substrate for short-term habituation. It seems more likely that when a stimulus representation enters the refractory state, it is due to a reduction in the excitability of the appropriate neuronal ensemble. Consistent with this hypothesis there is evidence from human fMRI studies that neuronal activity is reduced when stimuli are repeated, compared with when a novel stimulus is presented (Henson et al.,^2003^; Ranganath and Rainer,^2003^; Grill-Spector et al.,^2006^; Kumaran and Maguire,^2006^). Furthermore, it has been found in rats that neurons in the perirhinal cortex reduce their firing to repetitions of stimuli (Brown and Aggleton,^2001^). Therefore, GluA1 may play an important role in the short-term, recency-dependent reduction of neuronal excitability following a stimulus presentation (for a discussion see Sanderson et al.,^2010^), although the mechanism by which this might occur is not known. Nevertheless, regardless of the mechanism, it is clear that there are separate GluA1-dependent and GluA1-independent processes that contribute to the neural basis of stimulus-specific habituation.

As mentioned in Spatial Novelty Preference, the spatial novelty preference task is analagous in design to the spontaneous object recognition task in rodents (Ennaceur and Delacour,^1988^). Similar to the present results, examining a form of spatial recognition memory, there is evidence that short-term and long-term perirhinal-dependent object recognition memory can be dissociated from one another (Barker et al.,^2006^). In contrast to our analysis of spatial recognition memory in terms of a dual-process account of habituation, it has been suggested that recognition memory does not reflect habituation (Brown,^1996^; Brown and Xiang,^1998^). This is due to neurons in the perirhinal cortex, a brain area essential for object recognition memory, showing a pattern of repetition suppression effects that differ from those typically seen in studies examing the neural basis of unconditioned response habituation (Thompson and Spencer,^1966^; Horn,^1967^; Kandel,^1981^). Given that we suggest that habituation can be caused by multiple processes, it is possible that these separate processes may determine the nature of the neuronal repetition suppression. It is also likely that differences in the repetition suppression effect may depend on many other factors such as the nature of the stimulus, stimulus intensity, and the interval between stimulus presentations. What is common throughout these studies is that repeated presentation of a stimulus results in a reduction of neuronal responding. By applying Wagner's (1981) model to spontaneous recognition memory in rodents it may be possible to derive novel predictions concerning the underlying neuronal responses. For example, the neuronal suppression to a stimulus may depend on whether memory is associatively evoked or directly activated by a recent presentation of the stimulus.

## THE FRONTAL CORTEX AND ALTERNATION BEHAVIOR

Although both the hippocampus and the frontal cortex have been argued to play a role in short-term or working memory (Baddeley and Hitch,^1974^; Olton et al.,^1979^; Goldman-Rakic,^1987^) the frontal cortex may not always be necessary for alternation performance in rodents, or at least may only be required under certain conditions. For example, some studies fail to find effects of frontal lesions on alternation (Aggleton et al.,^1995^; Deacon et al.,^2003^), or where effects have been found, the deficits are often small and transient (Shaw and Aggleton,^1993^; Sanchez-Santed et al.,^1997^; Delatour and Gisquet-Verrier,^2000^; Dias and Aggleton,^2000^; Walton et al.,^2003^; Kellendonk et al.,^2006^; Mariano et al.,^2009^). While it has been argued that the frontal cortex may be important when delays are introduced between the sample and choice trial (Seamans et al.,^1995^; Floresco et al.,^1997^; Taylor et al.,^2003^; Di Pietro et al.,^2004^), rodents with medial prefrontal cortex lesions can also show high levels of alternation at long delays (Gisquet-Verrier and Delatour,^2006^). It has been argued that this may reflect that the medial prefrontal cortex is not necessary for alternation per se, but may be required when unexpected changes in testing procedure cause interference in performance (Gisquet-Verrier and Delatour,^2006^). Thus, the effects of frontal damage on alternation contrast markedly with the effects of hippocampal lesions.

The fact that the frontal cortex is important for working memory in humans (Baddeley and Hitch,^1974^; Baddeley,^1992^), but may not always be necessary for alternation behavior in rodents, further suggests that alternation behavior is unlikely to reflect active maintenance of information (i.e., working memory). As we have suggested, alternation behavior need only reflect short-term habituation of responding to stimuli on the basis of their relative familiarity as a result of recent exposure. In rodents, the role of the frontal cortex has been implicated in more complex forms of goal directed behavior (e.g., Ragozzino et al.,^1999^; Haddon and Killcross,^2005^; de Wit et al.,^2006^; Marquis et al.,^2007^; Dwyer et al.,^2009^) that may require active maintenance of information (Miller and Cohen,^2001^). The utilization of short-term memory traces in these more complex tasks is clearly different from the processes involved in short-term habituation.

## THE PROBLEM FOR ANIMAL MODELS OF WORKING MEMORY DISORDERS

Since Olton's description of spatial alternation, win-shift behavior as a form of working memory, spatial working memory tests have been widely used to test animal models of disorders that cause disruption to working memory in humans. One such disorder is schizophrenia that has been closely associated with deficits in working memory (e.g., Goldman-Rakic,^1994^). Also, the fact that schizophrenia is associated with both neuropathology in the hippocampus (Harrison,^2004^) and disrupted working memory in humans has strengthened the argument for examining hippocampus-dependent spatial working memory in rodents. Thus, spatial working memory tasks in rodents have been widely used as a plausible indicator of a schizophrenic phenotype. Our analysis of alternation tasks suggests that they are not necessarily a model of human working memory, but an example of stimulus-specific, short-term habituation. It is clear that short-term habituation in rodents requires a psychological process that is distinct from those involved in human working memory tasks such as the *n*-back or reverse digit span task. Therefore, results with spatial working memory tasks in rodents may be misleading in terms of modeling human working memory.

While alternation may not be a model of working memory in humans, the fact that it requires short-term habituation may demonstrate that it requires other psychological processes that are analogous to those that are disrupted in specific disorders like schizophrenia. For example, habituation is merely the consequence of a reduction of processing that a stimulus can receive. Therefore, habituation may occur because the attention paid to a given stimulus decreases. Thus, alternation behavior in rodents may reflect aspects of attentional processes in humans and thus relate to attentional deficits in schizophrenia (e.g., prepulse inhibition, Braff et al.,^1978^; Hazlett et al.,^2007^). Tests of stimulus-specific habituation (e.g., alternation behavior, object recognition) may provide a simple means of assessing reductions in attention paid to stimuli.

## CONCLUSIONS

Although alternation behavior is considered a demonstration of working memory in rodents, our recent evidence from GluA1^−/−^ mice provides an alternative analysis. We argue that the pattern of results suggest that alternation performance relies on short-term habituation to spatial stimuli. The hypothesis that GluA1 is necessary for short-term habituation can account both for why GluA1^−/−^ mice are impaired on alternation tasks and why, under some circumstances they can show enhanced spatial learning. The characterization of alternation behavior as a form of short-term habituation can aid translation between animal and human work in neuropsychological disorders.
